# miRNA: The nemesis of gastric cancer (Review)

**DOI:** 10.3892/ol.2013.1428

**Published:** 2013-06-26

**Authors:** XIAOHUI XU, XIAODONG YANG, CHUNGEN XING, SHUYU ZHANG, JIANPING CAO

**Affiliations:** 1 Department of General Surgery, The Second Affiliated Hospital of Soochow University, Suzhou 215004, P.R. China; 2 School of Radiation Medicine and Protection, Medical College of Soochow University, Suzhou 215123, P.R. China

**Keywords:** miRNA, gastric cancer, serum, plasma, biomarker, exosome

## Abstract

microRNAs (miRNAs) are a group of small non-coding RNAs that are ~22 (18 to 25) nucleotides (nt) long and have been associated with a variety of diseases, including cancer. Increasing evidence indicates that miRNAs are essential in the development, diagnosis, treatment and prognosis of a variety of tumors. The utility of miRNAs as biomarkers for diagnosis and of target molecules for the treatment of cancers is increasingly being recognized. With the discovery of circulating miRNAs, a non-invasive approach for the diagnosis and treatment of cancer has been identified. This review summarizes the role of miRNAs in the development of different tumors, as well as a variety of other biological events. Moreover, this review focuses on analyzing the function and mechanism of gastric cancer-related miRNAs and investigates the importance of circulating miRNAs in gastric cancer, as well as their origin. Finally, this review lists a number of the problems that must be solved prior to miRNAs being used as reliable non-invasive tools for the diagnosis, treatment and prognosis of gastric cancer.

## 1. Introduction

The incidence of gastric cancer ranks fourth in males and fifth in females, while the mortality ranks third in males and fifth in females among all cancers worldwide (1). The incidence and mortality of gastric cancer in developing countries in males and females are much higher compared with developed countries (1). With improvements in diagnostic and therapeutic technology, the survival time of patients with early gastric cancer has been greatly extended. However, the prospects for the treatment of patients with advanced gastric cancer are not optimistic. At present, there are a number of non-surgical treatment approaches, although surgery remains the primary method for the radical treatment of gastric cancer.

microRNAs (miRNAs) are becoming increasingly recognized as important in cancers, including gastric cancer. To date, >1,400 miRNAs have been described in humans (2). Yu *et al* observed that in gastric cancer cell lines 17 miRNAs were upregulated and 146 miRNAs were downregulated compared with normal gastric mucosa (3). miRNAs specific to the tumorigenesis and development of tumors have become hot spots for cancer research. miRNAs are associated with the development of tumor biology, as well as the diagnosis, treatment and detection of prognosis of tumors. In addition, the reliability of miRNA detection in the circulatory system of tumor patients has also been demonstrated. One study showed that miRNA expression is closely associated with the tumorigenesis, progression and prognosis of gastric cancer (4), and the detection of miRNA in the circulating system may offer new biomarkers for gastric cancer (5). Consequently, detecting miRNAs in the serum/plasma may become a non-invasive pathway for providing a basis for the treatment, diagnosis and prognosis of gastric cancer.

## 2. Discovery of miRNAs

In 1993, Victor Ambros first identified miRNA in *C. elegans*(6). Simultaneously, Gary Ruvkun identified the gene Lin-14 as the first miRNA target (7). These two important findings demonstrated a new post-transcriptional gene regulation mechanism. Approximately seven years later, the importance of miRNAs was realized when Ruvkun and Horvitz identified another miRNA in *C. elegans*(8), and when interest was focused on another short-chain RNA, small interfering RNA (siRNA; involved in the process of RNA interference and related phenomena in plants and animals). In 2001, three research groups from different countries all identified 21–22 nucleotide (nt) non-coding small RNA molecules in *C. elegans*, *Drosophila* and the human body (9). These single-stranded small RNA molecules with spatial and temporal expression were different from the previously reported siRNA detected in the interference pathway (RNA interference; RNAi) and were subsequently named miRNA.

## 3. Biosynthesis and biological function of miRNAs

miRNAs are encoded by specific miRNA genes in the genomic DNA, which are first transcribed by RNA polymerase II into a stem-loop that is ~500 to 3,000 bp long (10). This initial transcript is known as pri-miRNA. These pri-miRNAs in the nucleus are further transformed into ~60 to 70-nt hairpin-shaped precursor miRNAs (pre-miRNAs) by the Drosha enzyme (a type of RNase III enzyme). Precision processing of the pre-miRNA is extremely important as this produces the ‘seed’ region of miRNA, which is the determining factor leading to gene silencing. This ‘seed’ region is the target area on the mRNA that is complementary to the 2–7 positions of the antisense miRNA oligonucleotide. The pre-miRNAs are exported into the cytoplasm by Exportin-5 (Exp-5) and cleaved by Dicer to generate 20–24 nt RNA duplexes, one strand of which is loaded into the Argonaute-containing RNA-induced silencing complex (RISC). miRNA-RISC complexes are able to silence target mRNAs via imperfect complementarity with sequences located in the 5′-UTR, coding sequences and, most commonly, the 3′-UTR (11). When pre-miRNA is transported to the cytoplasm by the transport protein Exp-5, the pre-miRNA hairpin in the cytoplasm is cleaved into two strands by Dicer (RNase III), with the release of two complementary 22-base nt chains. Subsequent to being processed by the enzyme Dicer, the antisense miRNA chain and mRNA target region complementary base pair together to form a complex with Argonaut proteins. Argonaut guides the miRNA chain to reach the target sequence on the mRNA and combines with RISC (12,13). RISC combines with target mRNA at the 3′-UTR and the miRNA base pairs with the target mRNA, causing target mRNA cleavage and inhibition of protein expression (13–17).

miRNAs regulate gene expression at the post-transcriptional level (18–20). Furthermore, miRNAs are members of the small RNA family, which includes small nuclear RNA (snRNA) involved in mRNA splicing (21), small nucleolar RNAs (snoRNAs), which directly modify ribosomal RNA (22), and siRNAs, which are generated by long double-stranded RNA precursors. siRNAs also functionally regulate gene expression, similar to miRNAs (23). miRNAs are highly-conserved endogenous RNA molecules that occur in the genome of animals, plants, fungi and viruses (24). It is currently estimated that miRNAs account for ~1% of the human genome (25) and they have been demonstrated to play crucial roles in the human body, including roles in growth (8), cell proliferation and cell apoptosis (26,27).

## 4. miRNAs and cancer

### Tumor-specific miRNAs

miRNAs have been associated with a variety of diseases, including cancer. Increasing evidence has demonstrated the importance of miRNAs in regulating biological characteristics common to various tumors, including self-growth signals, insensitivity to anti-growth signals, abnormal apoptosis, unlimited replication potential, sustained induction of angiogenesis and invasion and metastasis organization (28). Numerous researchers have identified tumor-specific miRNA signatures that accurately distinguish malignant tumors from various types of benign tissues, and have demonstrated that certain miRNAs are carcinogenic depending on the other gene mutations in the tumors (29). miRNA regulation in tumor cell lines directly affects cell proliferation and apoptosis, and similarly, a number of studies have identified a link between abnormal miRNA expression and intracellular signal transduction pathway abnormalities, as well as tumorigenesis (30–34). For example, miR-9 is activated by YC/MYCN, which induces cancer metastasis by regulating the metastasis suppressor protein E-cadherin (35), while miR-449a causes retinoblastoma (Rb)-dependent cell cycle arrest and cellular senescence in prostate cancer (36). Table I provides an overview of the expression of the same miRNAs in different tumor cells, the expression of different miRNAs in the same tumor cells and the associated target proteins and/or target genes of the miRNAs (35–48).

miRNAs constitute a new class of molecules that are able to promote the formation of cancer through interactions with oncogenes and/or tumor suppressor genes (49). However, different miRNAs have different signaling pathways and the target proteins/genes through which they affect the biological changes of cancer.

### Circulating miRNAs in cancer

The aforementioned examples show that miRNAs have the potential to function as biomarkers for the diagnosis, treatment and prognosis of cancer, although miRNA expression profiling may not be used without a tumor biopsy. Therefore, the identification of non-invasive methods for the diagnosis and treatment of cancer has become a goal for cancer research. A number of studies have proposed that the characteristics of the cancer genome, genetic and epigenetic, may be detected in the serum and plasma of cancer patients (50). Therefore, the detection of plasma miRNA is a useful approach for the diagnosis, treatment and monitoring of the prognosis for cancer patients. miRNAs are involved in the development and differentiation of tumors and the lineage-dependent mechanism. Consequently, this becomes the basis for tumor development. Since miRNA detection by PCR is highly sensitive and of low complexity compared with protein detection, modifications of miRNAs are possible and synthetic high-affinity miRNA “capture” reagents are available, miRNAs may have a high value as biomarkers. In addition, circulating miRNAs in human blood and other body fluids are plentiful, have remarkable stability and may be measured in routine clinical diagnosis. Therefore, circulating miRNAs are considered to be extremely promising cancer biomarkers (51–53). At present, an increasing number of scholars are attempting to identify more specific and highly sensitive miRNAs from the plasma of various tumor patients for use as non-invasive biomarkers for the diagnosis, treatment and prognostic evaluation of tumors. Such a simple, feasible and acceptable method is likely to optimize the therapeutic effect for patients. In recent years, there are have been a number of studies on plasma miRNAs as biomarkers. Lawrie *et al* identified the first circulating miRNA in 2008 (54). The study observed that miR-21 expression was associated with the relapse-free survival of patients with diffuse large B-cell lymphoma (DLBCL) and suggested that miRNAs have potential as non-invasive diagnostic markers for DLBCL and possibly other cancers (54). Subsequently, the plasma-based detection of miRNA has become a research hot spot. Table II provides an overview of the functions of plasma or serum miRNAs in various types of cancer (55–64).

All current research results show that miRNAs have promise as novel non-invasive biomarkers for the elimination of false-positive and -negative results in routine testing. However, the source of the circulating miRNAs and how these circulating miRNAs function must be identified for the proper use of circulating miRNA biomarkers in evidence-based medicine (65).

## 5. miRNAs and gastric cancer

Since miRNAs have been shown to be significant factors during the progression of gastric cancer, global miRNA expression profiles have been performed using microarrays, real-time PCR or next-generation sequencing approaches. A significant number of the functions and mechanisms of miRNAs in the process of the development of gastric cancer have been identified.

### Biological effects of miRNAs in gastric cancer

The incidence of gastric cancer is the result of interactions between *Helicobacter pylori*, genetic predisposition, eating habits and environmental factors, although oncogenes and tumor suppressor genes eventually determine gastric carcinogenesis. However, miRNAs are not only involved in oncogene-induced tumors, but also in tumors caused by tumor suppressor genes; their role in gastric carcinogenesis complements and enriches the mechanism of tumorigenesis. Table III provides an overview of the roles of various miRNAs in the proliferation of gastric cancer (66–82). Considerable progress has also been made in research into the mechanism of action of miRNAs in gastric cancer cell invasion and metastasis. Table IV provides an overview of the role of various miRNAs in the invasion and metastasis of gastric cancer (83–90).

miRNAs are pivotal in the development of gastric cancer. It is of note that certain miRNAs do not function alone and various clusters of miRNAs share functional associations. Therefore, it is extremely important to clarify whether their role is independent or synergistic with other miRNAs when the biological functions of certain clusters of miRNAs are studied.

### miRNAs and gastric cancer pathology

The detection of specific miRNAs may become a potential field in marker development (91). Studies have shown that different tumors have specific miRNA expression patterns and that a variety of tumors have specific miRNA expression profiles (92). A comparative analysis of miRNA expression profiles between tumors and normal tissue suggests a new approach for the genetic diagnosis of tumors. At present, the role of miRNAs in the diagnosis and treatment of gastric cancer has increased significantly worldwide. miR-21 appears to be important in tumorigenesis due to its upregulation in almost all types of human cancer. Furthermore miR-21 is overexpressed in 92% (34/37) of gastric cancer samples and is thus considered to be a promising novel biomarker for gastric cancer, as well as lymph node metastasis (93,94). In gastric cancer, upregulated miRNA-106a and miR-143, as well as downregulated miR-203, are associated with gastric tumor size, stage, lymph nodes and distant metastasis. Consequently, they may become a new type of potential biological diagnostic molecule for gastric cancer (95–97). A study has shown that the reduced expression of miR-574–3p occurs mainly in the early stages of gastric cancer or in cancers with a high level of differentiation, suggesting that it may be used as a marker for mild cases of gastric cancer (98).

Therefore, miRNAs are important in pathological staging, lymph node metastasis and distant metastasis of gastric cancer. If miRNA changes specific to the development of gastric cancer are identified and the stage of gastric cancer is determined, this approach may become a pathway for aiding in the diagnosis of gastric cancer by detecting this type of miRNA. However, further important information for the clinical diagnosis of gastric cancer should be provided and more clinical data must be collected to determine the feasibility.

### miRNAs and gastric cancer diagnosis and treatment

At present, the main methods of treatment for gastric cancer are surgery, chemotherapy, radiotherapy and biologically targeted therapy, of which surgery is the most important. In numerous cases, a tumor diagnostic tool also provides a therapeutic approach. There are a number of studies on gastric cancer treatment through the targeting of miRNA. For example, cell proliferation, migration and invasion in gastric cancer cells have been demonstrated to be significantly increased following miR-181b transfection, so miR-181b may be a potential molecular target for anticancer therapeutics in gastric cancer (99). Zhao *et al* revealed that miR-7 functions as an anti-metastatic microRNA in gastric cancer by targeting the insulin-like growth factor-1 receptor (100). Targeting the miR-7/IGF1R/Snail axis may be useful as a therapeutic approach for blocking gastric cancer metastasis. miRNAs produce a negative regulation of proteins by combining with the target mRNA, and it is possible to take advantage of their features to up- or downregulate the expression of key tumor-associated genes for therapeutic purposes. miRNAs, either as oncogenes or tumor suppressor genes, regulate the biological characteristics of gastric cancer. Inhibiting miRNAs as oncogenes or increasing miRNAs as tumor suppressor genes using antisense oligonucleotides and siRNA technology presents a new approach to biological therapy for gastric cancer. The identification of specific tumor-associated miRNA coding genes provides new targets for cancer gene therapy. Studies to identify drug targets for miRNAs, as well as small molecule compounds that are able to inhibit miRNA activity, are being conducted by predicting three-dimensional structures of the miRNA (101). Certain studies have investigated the use of anti-miRNA antisense oligonucleotides (AMOs) to reduce or knock out overexpressed tumor-associated miRNAs, producing tumor suppressor effects. The knockout of miR-21 by AMOS causes gastric cancer cell proliferation to be reduced significantly and apoptosis to increase significantly (67). The downregulation of prohibitin by miR-27a may explain why the suppression of miR-27a inhibits gastric cancer cell growth, further supporting the hypothesis that miR-27a functions as an oncogene (102). The inhibition of miR-421 expression has been shown to decrease the growth of MGC-803 and SGC-7901 gastric cancer cells *in vitro*, upregulating the expression of its cancer-associated target genes, CBX7 and RBMXL1 (103). By contrast, the expression of tumor suppressor miRNA, induced by miRNA mimics and delivered by virus or liposome, gradually reduced the growth of gastric tumors (104). miR-34 is has been demonstrated to be involved in the downstream p53 pathway and is a potential tumor suppressor of the target genes Notch, HMGA2 and Bcl-2, which are involved in cancer stem cell self-renewal and survival (105). The miR-34 mimics or infection with the lentivirus function as miR-34. A study has shown that miR-34 is able to impair cell growth, cause the accumulation of the cells in the G_1_ phase, increase caspase-3 activation and more significantly, inhibit tumorsphere formation and growth, indicating that the restoration of the tumor-suppressor, miR-34, may provide a novel molecular therapy for p53-mutant gastric cancer (106). Resistance to chemotherapy remains one of the main obstacles for improving the overall survival and quality of life of patients with gastric cancer. Treatment with miRNA may be used as a regulatory tool for tumor cells in the chemotherapy response. Xia *et al* suggested that miRNAs may be involved in the development of multidrug resistance (MDR) in gastric cancer cells (107). miR-15b and miR-16 are able to modulate the sensitivity of gastric cancer cells to certain anticancer drugs, at least in part, by regulating BCL2 expression (108). So far, the potential of miRNA-based treatment for malignant disease remains largely to be developed. In addition to the previously mentioned research areas, miRNA remains to be investigated in tumor metastasis, angiogenesis and radiation resistance.

Before the application of miRNA-based therapy may occur, there are several major obstacles to overcome. First, miRNAs have multiple targets, producing the risk of accidental off-target effects, and therefore miRNA-based therapy requires careful assessment. Second, the expression of target genes is controlled by several different miRNAs, which may reduce the effect of miRNA-based therapy. Finally, there is a lack of a sufficiently specific and effective transport system for miRNA (109).

Although miRNA-targeted treatment is likely to provide a new approach for the treatment of gastric cancer, effective treatment may require a combinatorial approach to target multiple oncogenic miRNA clusters. If good use is made of multi-targeted miRNA in treatment, particularly when adaptive resistance occurs, it may produce therapeutic effects via a variety of alternative pathways in tumor cells. However, there is a long way to go before miRNAs are suitable as a therapeutic target for clinical application.

### miRNAs and gastric cancer prognosis

The prognosis of patients subsequent to treatment is important for each patient and their families. In recent years, attempts have been made to identify prompt detection methods for the prognosis of gastric cancer using the fields of biochemistry, immunohistochemistry and genetic testing (110,111), although a reliable, specific, sensitive and practical detection method has yet to be detected. However, it has been possible to identify a new method for detecting the prognosis of gastric cancer patients through the study of miRNAs. Yu *et al* and Chen *et al* detected high expression levels of miR-93 in gastric cancer, particularly in advanced and metastatic cases (3,103). The authors suggested that miR-93 may be critical in the carcinogenesis of gastric cancer. Notably, miR-93, as well as miR-1, may predict a poor survival outcome in gastric cancer patients (3,112,113). Furthermore, Tsai *et al* demonstrated that miR-196a may be a novel biomarker for detecting gastric cancer and monitoring disease recurrence (114). Multivariate analyses have indicated that low miR-125a-3p expression is an independent prognostic factor for survival, while *in vitro* assays have demonstrated that miR-125a-3p suppresses the proliferation of gastric cancer cells (115).

Therefore, the continuous investigation of gastric cancer-associated miRNAs may lead them to become new assessment biomarkers following treatment for gastric cancer. These biomarkers may not only increase the effectiveness of the evaluation but may also become the main basis of this evaluation.

## 6. Serum/plasma miRNAs as biomarkers/signaling molecules for gastric cancer

At present, the majority of the discoveries of gastric cancer-associated miRNAs have been based on gastric carcinoma and adjacent normal tissues. Since this is an invasive approach, it is a significant request to make of patients and not one that is readily accepted. However, great progress has been made in investigating whether serum and plasma miRNAs may be used as non-invasive biomarkers for the diagnosis, treatment and detection of prognosis in gastric cancer patients. Tsujiura *et al* published a study demonstrating that plasma miRNA assays have several potential clinical uses, including for screening patients at a high risk of gastric cancer and monitoring disease recurrence during the follow-up period after gastrectomy (5). These miRNA biomarkers may be powerful and useful for confirming the completeness of tumor resection and evaluating the efficacy of adjuvant therapies if the clearance of plasma miRNAs may be elucidated (5). In addition, Konishi *et al* observed that plasma miR-451 and miR-486 may be useful blood-based biomarkers for the screening of gastric cancer. The authors noted that the levels of these two miRNAs decrease in post-operative plasma in 90 and 93% of patients, respectively. However, in comparison with healthy controls, the levels of the two miRNAs were demonstrated to be significantly higher in the plasma of post-operative patients (116). The results of further studies have shown that the levels of circulating miR-17–5p/20a may be a promising non-invasive molecular marker for pathological progression, predicting prognosis and monitoring chemotherapeutic effects for gastric cancer (117). Studies have also shown that serum miR-221, miR-376c, miR-744 and miR-378 have clear potential as novel non-invasive biomarkers for the early detection of gastric cancer, and that miR-378 may be used as a valuable biomarker alone (118,119). These findings have pioneered a new approach for the diagnosis and treatment of gastric cancer and demonstrated that miRNAs are likely to become a routine examination indicator for the development and prognosis of gastric cancer, similar to CA199, p53 or other tumor markers.

As mentioned previously, miRNAs are extremely stable in blood plasma and serum (56). Consequently, this makes miRNA levels as suitable for testing for clinical diagnosis as tumor markers and biomarkers. The source of the miRNAs circulating in blood samples remains unclear. Chim *et al* suggested two hypotheses for this: The presence of circulating miRNAs may be due to tumor cell death and lysis or they may be released by tumor cells into the extracellular microenvironment of blood vessels (120). Ohshima *et al* demonstrated that the amount of total RNA in culture media was relatively higher in the NCI-H69, Lu-135 and Colo205 cell lines compared with in the exosomes, which was consistent with the ‘tumor cell death’ hypothesis. However, the authors also revealed that the let-7 miRNA family is selectively secreted into serum and plasma via exosomes in metastatic gastric cancer cell lines (121), while Tanaka *et al* demonstrated that the level of miR-92a was decreased in the blood plasma of acute leukemia patients while remaining high in the tissue samples (121). These results demonstrated that miRNAs may serve as signaling molecules transferred by exosomes between tumor cells and serum/plasma.

Most significantly, circulating miRNAs are not only tumor biomarkers for gastric cancer, but also signaling molecules involved in the proliferation, tumorigenesis and metastasis of gastric cancer.

## 7. Conclusions

In summary, increasing attention is being paid to miRNAs in gastric cancer. To identify the best approach for the diagnosis, treatment and evaluation of the prognosis of gastric cancer via miRNAs, the detection of serum/plasma miRNAs is likely to be the first choice. However, to make this a reality, breakthroughs are required in the following areas: i) Determining the source of circulating miRNAs; ii) validating a classical pathway of circulating miRNA transmittance; iii) clarifying the mechanism of the action of various circulating miRNAs in gastric cancer; iv) determining how the circulating miRNAs in different stages of gastric cancer development are expressed and how miRNAs develop in this development process; v) highly specific and sensitive detection of miRNA pre- and post-operatively, as well as at various stages of gastric cancer; vi) clarifying the mechanism of synergy/antagonism of various circulating miRNAs in the occurrence and development of gastric cancer; and vii) determining the feasibility of certain miRNA target therapies at different stages of gastric cancer according to associated miRNA specificity.

## Figures and Tables

**Table I:** Tumor-associated miRNAs and their targets.

miRNA	Up- or down-regulation	Target gene(s)	Target proteins	Cancer	References
miR-9	Up	YC/MYCN	E-cadherin	Breast cancer cells	(35)
miR-449a	Down	Rb	-	Prostate cancer cells	(36)
miR-223	Down	E2F1	C/EBPα	Acute myeloid leukemia	(37)
miR-34a and miR-34b/c	Down	P53	CDK4/6, Cyclin E2, MET and Bcl-2	Neuroblastoma, colorectal, lung, pancreatic, breast and ovarian cancer, bladder and renal cell carcinoma, liver carcinoma, oral squamous cell carcinoma,	(38,39)
miR-21	Up	Pdcd4	u-PAR	Colon cancer	(40,41)
miR-17–92	Up	K-Ras and c-Myc	Tsp1 and CTGF	Colon cancer	(42)
miR-145	Down	TP53	ER-α	Breast cancer cells	(43)
miR- 483-3 and miR-421	Up	DPC4/Smad4	DPC4/Smad4	Pancreatic cancer	(44,45)
miR-224	Up	CDC42, CDH1, PAK2, BCL-2 and MAPK1	CDC42, CDH1, PAK2, BCL-2 and MAPK1	Hepatocellular carcinoma	(46)
miR-106b-25	Up	Caspase-7	Focal adhesion	Prostate cancer	(47)
miR-34	Down	Met and Bcl-2	p53	Lung adenocarcinoma	(48)

miRNA, microRNA; Rb, retinoblastoma.

**Table II:** Functions of plasma or serum miRNAs in different cancer.

miRNA	Serum/plasma	AUC	Sensitivity (%)	Specificity (%)	Functions	Cancer	References
miR-21	Plasma	0.63	64	89	Discriminate cancer or normal	Pancreatic ductal adenocarcinoma	(55)
miR-210		0.62					
miR-155		0.60					
miR-196a		0.66					
miR-141	Serum	0.88	60	100	Discriminate cancer or normal	Prostate cancer	(56)
miR-141	Plasma	0.88	77	90	Relevant to tumor stage	Colorectal cancer stage I–III	(57)
			91	77		Colorectal cancer stage IV	
miR-92	Plasma	0.88	89	70	Discriminate cancer or normal	Colorectal cancer	(58)
miR-29a and miR-92a	Plasma	0.88	83	85	Discriminate cancer or normal	Advanced colorectal cancer	(59)
microRNA-29a	Serum	0.88	75	75	Relevant to liver metastasis	Colorectal cancer	(60,61)
miR-221	Plasma	0.88	86	41	Relevant to plasma level of miR-221 and p53	Colorectal cancer	(62)
miR-15b and miR-27b	Serum	0.98	100	84	Discriminate cancer or normal	Non-small cell lung cancer	(63)
miR-125b	Serum	0.66	96	38	Relevant to poor prognosis	Non-small-cell lung cancer stage I–II	(64)
		0.84	93	66		Non-small-cell lung cancer stage III	
		0.90	95	67		Non-small-cell lung cancer stage IV	

AUC, area under the curve; miRNA, microRNA.

**Table III:** Role of different miRNAs in the proliferation of gastric cancer.

miRNA	Up- or down-regulation	Regulatory pathway	Inhibit or promote biological function	Related biological function	Detection method	Cell lines	References
miR-449	Up	Activating p53 pathway	Inhibit	Proliferation	Taqman miRNA assays	SNU638 and MKN74	(66)
miR-21	Up	PTEN expression	Promote	Proliferation and invasion	Real-time PCR	BGC-823	(67,68)
miR-9 and miR-433	Down	GRB2 and RAB34 expression	Promote	Proliferation	qRT-PCR PCR	SGC7901	(69)
miR-222	Up	p27 and p57	Promote	Proliferation	Real-time	SNU-638, AGS and MKN-28	(70)
miR-375	Up	PDK1/Akt signalling pathway	Inhibit	Proliferation	miRNA Microarray	NUGC3, AZ521 and MKN74	(71)
miR-375	Up	JAK2 oncogene PDK1	Inhibit	Proliferation	qRT-PCR	MGC-803, BGC-823, SGC-7901, et al	(72,73)
miR-9	Up	NF-κB1gene	Inhibit	Proliferation	Stem-loop RT-PCR	MGC803	(74)
miR-141	Down	FGFR2 signalling pathway	Promote	Proliferation	qRT-PCR	MGC-803, HGC-27, SGC-7901, et al	(75)
miR-199a	Up	Smad4TGF-β signalling pathway	Promote	Growth and survival	Real-time RT-PCR	SNU-16, AGS, BGC-823	(76)
miR-181a	Up	KLF6 gene	Promote	Proliferation, colony formation, migration, and invasion	qRT-PCR	SGC-7901	(77)
miR-181b	Up	CREB1	Inhibit	Proliferation and colony formation	-	-	(78)
miR-124	Up	SPHK1	Inhibit	Proliferation and tumourigenicity	qRT-PCR	MGC-803 and SGC-7901	(79)
miR-409-3p	Up	PHF10	Inhibit	Proliferation	qRT-PCR	SGC-7901	(80)
miR-182	Up	CREB1	Inhibit	Proliferation and colony formation	qRT-PCR	MGC-803, BGC-823 and SGC-7901	(81)
miR-223	Up	FBXW7/hCdc4 gene	Promote	Proliferation	Real-time RT-PCR	SGC7901	(82)

miRNA, microRNA; CREB1, cAMP responsive element binding protein 1; SPHK1, sphingosine kinase 1.

**Table IV:** Role of different miRNAs in the invasion and metastasis of gastric cancer.

miRNA	Up- or down- regulation	Regulatory pathway	Inhibit or promote biological function	Related biological function	Detection method	Cell lines	References
miR-409-3p	Up	RDX	Inhibit	Invasion and metastasis	RT-PCR	SGC-7901, MKN-45 and GES-1	(83)
miR-27	Up	EMT	Promote	Metastasis	RT-PCR	AGS	(84)
miR-625	Up	LIMS1-ILK-parvin	Inhibit	Migration and invasion	qRT-PCR	SGC-7901 and MKN-45	(85)
miR-625	Down	LIMS1-ILK-parvin	Promote	Migration and invasion	qRT-PCR	GES-1 and NCI-N87	(85)
miR-495 and miR-551a	Up	PRL-3	Inhibit	Migration and invasion	qRT-PCR	SGC7901, MKN45, MKN28, and BGC823	(86)
miR-10b	Up	HOXD10RhoC-AKT signaling pathway	Promote	Invasion	qRT-PCR	GES-1	(87)
miR-196b	Up	ETS2	Promote	Migration and invasion	qRT-PCR	AGS, HR and the embryonic kidney cell, 293T	(88)
miR-145	Up	N-cadherin protein	Inhibit	Invasion and metastasis	RT-PCR	GES-1 and NCI-N87	(89)
miR-610	Up	VASP	Inhibit	Migration and invasion	RT-PCR	BGC-823 and MKN-28	(90)

miRNA, microRNA.
